# Conceptual fluency increases recollection: behavioral and electrophysiological evidence

**DOI:** 10.3389/fnhum.2015.00377

**Published:** 2015-06-30

**Authors:** Wei Wang, Bingbing Li, Chuanji Gao, Huifang Xu, Chunyan Guo

**Affiliations:** Beijing Key Laboratory of Learning and Cognition, Department of Psychology, College of Education, Capital Normal UniversityBeijing, China

**Keywords:** conceptual fluency, recollection, recognition, event-related potential, N400

## Abstract

It is widely established that fluency can contribute to recognition memory. Previous studies have found that enhanced fluency increases familiarity, but not recollection. The present study was motivated by a previous finding that conceptual priming affected recollection. We used event-related potentials to investigate the electrophysiological correlates of these effects with conceptually related two-character Chinese words. We found that previous conceptual priming effects on conceptual fluency only increased the incidence of recollection responses. We also found that enhanced conceptual fluency was associated with N400 attenuation, which was also correlated with the behavioral indicator of recollection. These results suggest that the N400 effect might be related to the impact of conceptual fluency on recollection recognition. These study findings provide further evidence for the relationship between fluency and recollection.

## Introduction

Dual-process accounts of recognition memory posit that recognition memory performance is dependent on two distinct memory processes or types of memory, which are often referred to as recollection and familiarity. Recollection involves the retrieval of more information, such as contextual details associated with the study episode. In contrast, subjects experience familiarity with a recognition target when the target cues a feeling of memory, although they recall no additional confirmatory information (for a review, see Yonelinas, 2002). Neuropsychological (Yonelinas et al., 1998; Aggleton et al., 2005; Anderson et al., 2008), functional magnetic resonance imaging (fMRI; Brown and Aggleton, 2001; Davachi et al., 2003; Ranganath et al., 2004; Diana et al., 2007; Skinner and Fernandes, 2007), and event-related potential (ERP; for a review, see Rugg and Curran, 2007) studies have found that recollection and familiarity have distinct neural bases, which provides strong evidence for dual-process models.

Numerous ERP studies have suggested that familiarity and recollection are indicated by different ERP components, i.e., the FN400 and late positive component (LPC), respectively (Curran, 2000; Curran and Cleary, 2003; Mecklinger, 2006; Rugg and Curran, 2007). The FN400 is a negative ERP deflection with a 300–500 ms latency that is largest at frontal scalp locations and is more positive for old items relative to new items. The LPC is a posterior positive shift potential between 500 and 700 ms that is reduced for new items. However, some researchers have argued that FN400 potentials are associated with conceptual priming that co-occurs with familiarity (for a review, see Paller et al., 2007). For instance, Voss and Paller (2006) found that familiarity could be indicated by later posterior potentials, but that conceptual priming was associated with frontal potentials between 250 and 500 ms. Using squiggles as stimuli, Voss and Paller (2007) first dissociated conceptual implicit memory from familiarity during a recognition test. They emphasized that researchers should account for both implicit and explicit memory in their investigations into certain memory functions. The controversy over whether FN400 potentials are associated with familiarity or conceptual priming is related to the argument about whether FN400 has functional distinctions from N400. Voss and Federmeier (2011) directly contrasted the FN400 in recognition memory with the N400 in language and found no electrophysiological or functional difference. However, Bridger et al. (2012) argued that the experimental design used by Voss and Federmeier (2011) confounded recognition with semantic processes. When confounding factors were avoided, they found qualitative distinctions between FN400 and N400. The relationship between FN400 and N400 potentials remains an open topic.

Bearing implicit and explicit memory in mind, Stenberg et al. (2009) attempted to isolate conceptual priming from familiarity to examine the neural correlates of the latter. However, Lucas et al. (2010) insisted that their research procedures were problematic, although they had followed the right path. Indeed, dissociation of these two phenomena is difficult because the circumstances that produce conceptual fluency often yield familiarity. For instance, enhanced conceptual fluency may increase judgments of familiarity (Rajaram and Geraci, 2000; Wolk et al., 2004). In addition, multiple neurocognitive processes, including perceptual or conceptual fluency, can contribute to familiarity, and such contributions are dependent on various testing situations (Lucas et al., 2012; Taylor and Henson, 2012b; Lucas and Paller, 2013). A broader issue is involved here, i.e., whether implicit memory (e.g., conceptual priming) and explicit memory (e.g., recognition) are supported by multiple systems or a single system. Although abundant evidence indicates that implicit and explicit memory have distinct neural bases and rely on separate systems (for reviews, see Gabrieli, 1998; Squire, 2004), some researchers have suggested that dissociation evidence supporting the multiple-systems account can also be explained by the single-system account (Berry et al., 2006, 2008a,b, 2012). The boundary between implicit and explicit memory may not be clear cut (for a review, see Dew and Cabeza, 2011).

The remember/know (R/K) paradigm, which is based on subjective experience or varieties of consciousness, is often assumed to be a direct way to assess recollection and familiarity. Tulving (1985) introduced this paradigm in an attempt to assess whether retrieval occurred from episodic or semantic memory systems. The recognition memory processes that *R* and *K* responses reflect are debated. According to dual-process interpretations of the R/K paradigm, *R* and *K* responses reflect two qualitatively different memory processes (for a review, see Gardiner, 2001), e.g., recollection and familiarity, in recognition memory (Jacoby et al., 1997). In contrast, the signal detection theory of the R/K paradigm suggests that the distinction between *R* and *K* responses is quantitative, and that these responses reflect different confidence levels of memory retrieval (for a review, see Dunn, 2004).

The relationship between fluency, typically defined as the speed and ease of processing, and a subjective sense of familiarity, was first proposed by Jacoby and Dallas (1981), who theorized that stimuli experienced in the past were processed more fluently than novel stimuli. Thus, fluency was used as a heuristic in memory judgments. Accumulating evidence indicates that fluent stimuli are more likely to be endorsed as having been studied in a recognition test, and that illusions of recognition can be elicited by artificially enhancing fluency through priming or other means (e.g., Jacoby and Whitehouse, 1989; Goldinger et al., 1999; Whittlesea and Williams, 2001).

For example, in a pioneering study (Jacoby and Whitehouse, 1989), participants performed a word recognition memory test. In the test phase, presentation of a “context” word preceded the test words. In comparison with a condition in which no context word was presented, the probability of old responses was found to be increased when the context word matched the recognition test word (for studied and unstudied items). The authors explained this memory illusion in terms of unconscious influences on an attribution process. In the absence of awareness of its true source, participants were likely to erroneously attribute this increased fluency to the prior study phase. In support of this hypothesis, when the duration of primes was lengthened or participants were informed about them, the bias reversed such that primed test cues were less likely to be called old; this finding is consistent with participants' attribution of fluency to the primes, rather than to the study phase (e.g., Higham and Vokey, 2000; Klinger, 2001; Higham and Vokey, 2004).

Although Jacoby and Whitehouse's original findings did not specifically address the familiarity/recollection distinction, a subsequent study (Rajaram, 1993) using the “standard” R/K procedure showed that the recognition bias induced by masked primes increased only *K* judgments of studied and unstudied items. The author attributed this effect of repetition priming on familiarity to increased perceptual fluency. In a later study, Rajaram and Geraci (2000) used semantic primes and found that participants gave significantly more *K* judgments to items with related primes than to those with unrelated primes, while *R* responses were unaffected. These results suggest that the familiarity signal arises at the level of conceptual fluency. According to dual-process theories of recognition memory, familiarity is dependent on remembering specific items that are not associated with specific contextual markers, whereas recollection is dependent on remembering an association between an item and probably variable markers of the context in which it was encountered (for a review, see Yonelinas, 2002). Thus, subliminal priming could selectively enhance familiarity because it increases the fluency with which item representations, but not item-context representations, are activated. Substantial evidence suggests that fluency manipulations disproportionately influence familiarity, as opposed to recollection (Rajaram and Geraci, 2000; Miller et al., 2008; Woollams et al., 2008).

In a recent study, Taylor and Henson (2012a) used semantically related primes that were not associatively related in an attempt to isolate the effect of conceptual fluency on recognition memory judgments. When they included these conceptual primes with the standard repetition primes (with different blocks for each prime type), they found crossover interaction between prime type (conceptual *vs*. repetition) and memory judgment (*R vs*. *K*) on the priming effect: repetition primes increased *K* but not *R* judgments, and conceptual primes increased *R* but not *K* judgments. In an fMRI study, Taylor et al. (2013) replicated the previous behavioral finding that conceptual priming increased *R* judgments, and they found converging evidence for increased activity following conceptual primes in brain regions associated with recollection. Because the conceptual priming effect was not observed when participants were exposed only to conceptual primes, Taylor and Henson (2012a) posited that exposure to repetition primes might be a critical factor for conceptual fluency to increase *R* judgments. They proposed a “true recollection hypothesis” to explain the effect of conceptual priming on *R* hits in the fMRI study (Taylor et al., 2013).

However, neither repetition nor conceptual priming produced whole-brain effects in their study, which may have been due to the relative insensitivity of blood oxygen level–dependent signals to transient effects, such as very briefly presented masked primes. Thus, they were not able to compare neural correlates of these priming effects directly, as should be possible with ERPs because of their high temporal resolution. ERPs have been used widely to investigate the neural correlates of the masked priming effect during lexical decision tasks. Studies using the masked-repetition priming paradigm have shown that an earlier priming effect around the latency of P200 and a later priming effect around the latency of N400 are related to masked-repetition priming effects (e.g., Misra and Holcomb, 2003; Holcomb and Grainger, 2006). Studies using the Jacoby and Whitehouse paradigm in combination with ERPs have also been conducted to examine the electrophysiological correlates of fluency's contribution to recognition memory. For example, Woollams et al. (2008) found that masked repetition priming was associated with a central-focused ERP effect between 150 and 250 ms, and a posteriorly distributed ERP effect between 300 and 500 ms. However, Lucas et al. (2012) found only the posterior N400 ERP effect using the same paradigm. These two studies could not distinguish the electrophysiological correlates of perceptual and conceptual fluency because they used the masked-repetition priming paradigm. By investigating the electrophysiological correlates of masked conceptual priming, we could obtain the electrophysiological correlates of “true” conceptual fluency and investigate how it contributes to recognition memory.

In the current study, we used conceptually related two-character Chinese word pairs to investigate the effect of conceptual fluency on recognition memory in combination with electroencephalographic (EEG) recordings. In the study phase, participants were asked to perform an “interestingness” judgment task. In the test phase, half of the test words were conceptually primed (preceded by conceptually related words, here termed MCP trials) and half were unprimed (preceded by unrelated words, here termed MUP trials). Because the primes and targets had different perceptual features and were not associatively related, i.e., the targets were impossible free associates of the primes, any effect of MCPs could be attributed to conceptual fluency.

## Materials and methods

### Participants

Sixteen subjects (aged 20–26 years; 11 females) participated in the experiment. All were right handed and reported normal or corrected-to-normal vision. Each subject signed an informed consent form and received monetary compensation. This research was approved by the Human Research Ethics Committee at Capital Normal University.

### Materials

The stimuli consisted of 600 two-character Chinese word pairs that were conceptually related but not lexically associated (see Supplementary Material). Conceptual relatedness was defined according to the criteria of Taylor and Henson (2012a). Twenty-six college students evaluated conceptual relatedness and lexical association. Conceptual relatedness of each word pair was rated using a 10-point scale (10 = highly conceptually related, 1 = no conceptual relatedness). The forward and backward associations of each word pair were rated using a 10-point scale (10 = strongly lexical association, 1 = no lexical association). The mean conceptual relatedness score was 9.16. The mean scores of forward and backward association were 2.57 and 2.58. Mean strokes of primes and targets (sum of the two characters) were 16.9 and 17.1, respectively. Mean word frequencies of primes and targets were 53 per million and 51 per million, respectively. The frequency and number of strokes were matched in the MCP and MUP conditions. The old/new status and MCP/MUP status of the targets and primes were counterbalanced across participants. An additional 25 word pairs were used in filler trials. All items were presented in white against a black background.

### Procedure

The experiment consisted of five study-test blocks. In each study phase, 60 words were presented in random order, bounded by filler words (two primacy buffers and two recency buffers, ignored in the analysis). The participants were instructed to perform an “interestingness” judgment task by pressing one of two buttons to indicate whether or not the word was interesting.

In each test phase, the participants completed a recognition test in which 60 old words from the previous study phase were intermixed with 60 new words. Half of the trials in each test phase were MCP (i.e., an old or new word that was preceded by the masked presentation of a conceptually related word), and the remaining were MUP (i.e., an old or new word that was preceded by the masked presentation of an unrelated word). One forward mask (@@) and one backward mask (@@) sandwiched each prime word in the test phase. The participants were not informed about the presence of the masked words; they were told only that the forward mask symbols were used to “prompt” the appearance of the test words. It was emphasized that participants needed to focus on the test words to achieve the best memory performance.

**Figure 1:**
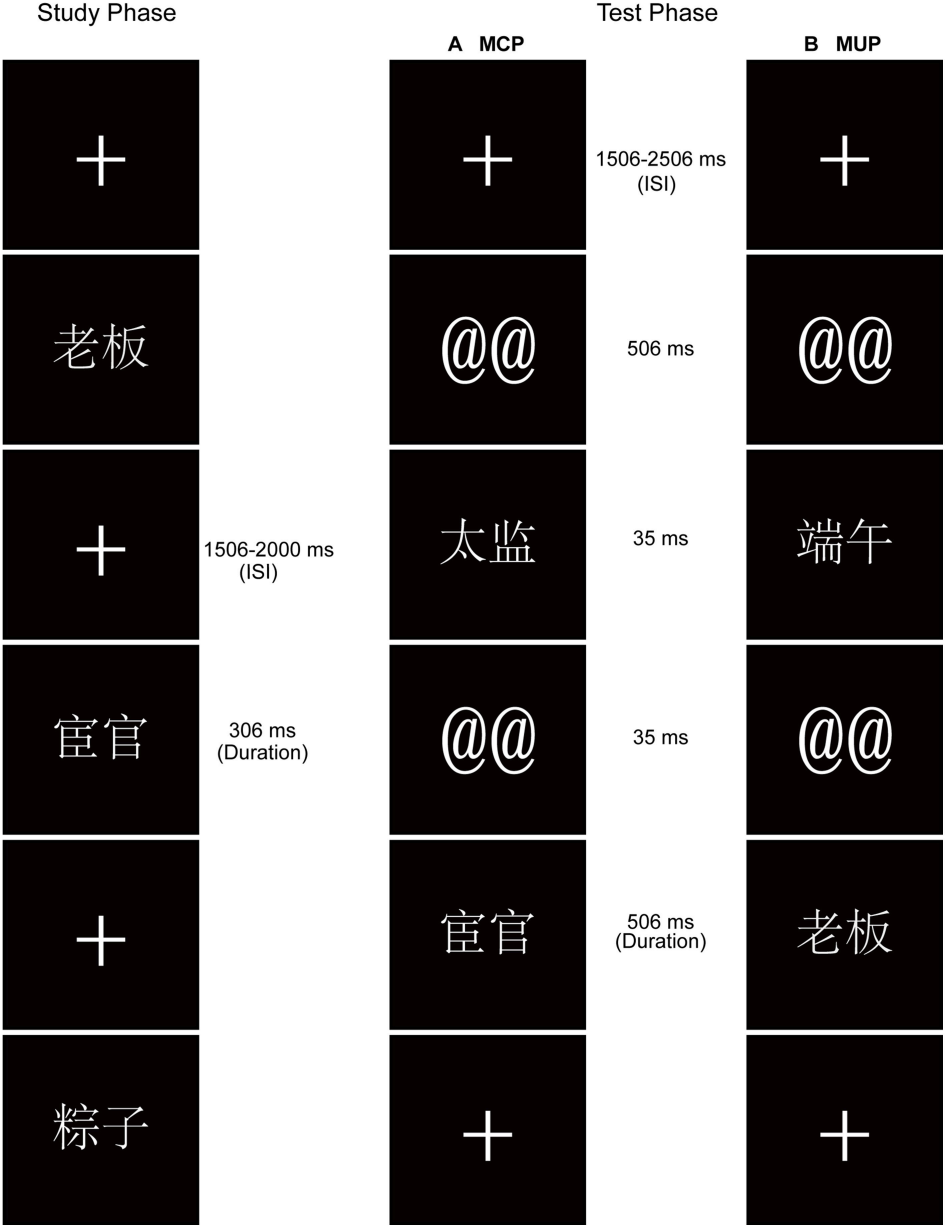
**Schematic representation of the experiment, showing examples of the stimuli**. In the study phase, participants performed an “interestingness” judgment task. The test-phase task was to make recognition judgments. **(A)** In masked conceptual priming (MCP) trials, an old or new word was preceded by masked presentation of a conceptually related word. **(B)** In masked unprimed (MUP) trials, an old or new word was preceded by masked presentation of an unrelated word.

Each study trial began with a fixation cross, followed by the display of a word for 306 ms and then a fixation cross. The interstimulus interval (ISI) was randomized to be between 1506 and 2000 ms. The test phase followed the study phase in each block, after a 30-s break during which the participants counted backward by threes. Each test phase was preceded by two practice trials (with one new and one old filler word; data not included in analyses). Each test trial began with the presentation of a fixation cross for a randomized duration of 1506–2506 ms; a forward mask was then presented for 506 ms, followed by the presentation of a conceptually related or unrelated word for 35 ms and then a backward mask for 35 ms. Next, a test word was presented for 506 ms, followed by a fixation cross (Figure 1). The participants were instructed to indicate using button presses whether they remembered seeing the test word (*R* judgment), whether the word was familiar (*K* judgment), or whether the word had not appeared in the previous study phase. Speed and accuracy of responses were emphasized. Note that we used the label “familiar,” rather than the traditionally “know” judgment, which was consistent with the terms used by Taylor and Henson (2012a). Recollection was defined as participants' retrieval of any contextual details from the study phase accomplished by recognition of a stimulus, such as recalling the “interestingness” judgment of the word or any other details from the learning episode (e.g., feelings about the word). Familiarity was defined as participants' belief that they had encountered the word previously, with no accompanying contextual detail.

Before the formal experiment, participants practiced distinguishing between recollection and familiarity in two short practice blocks, each of which contained 20 study and 40 test trials. To make sure that they had fully and correctly understood the instructions, participants were asked to report how *R* or *Familiar* judgments were made in some practice test trials. In particular, the experimenter ensured that participants did not equate *R*/*Familiar* responses with high/low confidence ratings. For example, they were instructed that a familiarity response was appropriate when they were sure (had high confidence) that the item had been presented in the study phase, but could recall no contextual detail.

After the five study-test blocks, participants were questioned about their awareness of the prime words. Only two of the 16 participants reported that they noticed words were presented between the “prompt” and the following test word in fewer than five trials in a test block, and that they were unrelated to the test word.

### Electrophysiological recording and analyses

ERPs were extracted from scalp EEG recordings from 62 Ag/AgCl electrodes embedded in an elastic cap. The electrode locations adhered to the extended international 10–20 system. The left mastoid was used as the reference site online. Signals were re-referenced offline to averaged mastoids. EEGs were recorded with a band pass of 0.05–100 Hz (0.05–40 Hz filtered offline), and sampled at a rate of 500 Hz. Impedance was less than 5 kΩ. Each 1000-ms averaging epoch began 100 ms prior to stimulus onset. Baseline corrections were performed using mean pre–stimulus onset amplitudes. Trials exceeding ±75 μV were rejected. EOG blink artifacts were corrected using a linear regression estimate. Repeated-measures analysis of variance (ANOVA) included Greenhouse-Geisser correction when necessary and Bonferroni-corrected *post-hoc* pairwise comparisons. The alpha level was 0.05.

## Results

### Behavioral results

The mean percentages of responses under each condition are presented in Table 1. For studied items, correct recognition included *R* hits and *K* hits. Responses to unstudied items were classified as false alarms (FAs) or correct rejections (CRs). The overall accuracy, computed as Pr (the proportion of hits minus the proportion of FAs, averaged across MCP and MUP trials), was 0.39 for *R* judgments and 0.27 for *K* judgments. For both types of recognition response, the accuracy was reliably greater than zero [*t*_(15)_ = 15.464, *p* < 0.001 for *R* judgments; *t*_(15)_ = 9.688, *p* < 0.001 for *K* judgments], indicating that memory was reflected to a degree greater than chance.

**Table 1:** **Mean percentage of responses in each condition**.

		**Remember**	**Know**	**New**
Studied	Conceptually primed	42.9 (2.7)	38.1 (2.4)	18.8 (1.8)
	Unprimed	40.6 (2.6)	39.1 (2.7)	20.1 (1.7)
Unstudied	Conceptually primed	2.4 (0.6)	12.3 (1.8)	85.2 (2.2)
	Unprimed	2.7 (0.6)	10.5 (1.6)	86.6 (2.0)

SE in parentheses.

To formally assess masked conceptual priming, a 2 (study status: studied/unstudied) × 2 (response type: *R*/*K*) × 2 (masked conceptual priming: MCP/MUP) ANOVA was performed for the percentage of old responses. Three-way interaction among study status, response type, and masked conceptual priming was significant [*F*_(1, 15)_ = 8.993, *p* = 0.009, *MSE* = 0.001].

Next, ANOVAs were performed for hits and FAs, respectively. A 2 (response type: *R*/*K*) × 2 (masked conceptual priming: MCP/MUP) ANOVA was performed for the hit rate. The interaction of response type with masked conceptual priming was significant [*F*_(1, 15)_ = 6.395, *p* = 0.023, *MSE* = 0.001]. Simple effect analysis revealed a significant difference between MCP and MUP data only for *R* responses [*F*_(1, 15)_ = 7.277, *p* = 0.017, *MSE* = 0.001].

The FA rate was analyzed similarly. The main effect of response type was significant [*F*_(1, 15)_ = 40.977, *p* < 0.001, *MSE* = 0.003], and the interaction of response type with masked conceptual priming was also significant [*F*_(1, 15)_ = 6.029, *p* = 0.027, *MSE* = 0.0003]. Simple effect analysis revealed that there was only a significant difference between MCP and MUP on the *K* responses [*F*_(1, 15)_ = 5.199, *p* = 0.038, *MSE* = 0.0005].

Overall, the masked conceptual priming effect selectively affected *R* hits and *K* FAs.

### ERP results

Analysis of the ERP results included three steps. First, ERPs from the test phase were analyzed without consideration of masked conceptual priming to determine ERP differences among *R* hits, *K* hits, and CRs. Second, to investigate the masked conceptual priming effects, we collapsed ERPs across response type and old/new status to examine overall differences between MCP and MUP trials. Third, masked conceptual priming effects were investigated across the three response types (*R* hit/*K* hit/CR).

Based on previous research on recognition memory and conceptual fluency (Paller et al., 2007; Lucas et al., 2012; Hou et al., 2013) and our observation of the results, the ERP amplitudes were averaged for three midline electrode clusters (frontal, F3/Fz/F4; central, C3/Cz/C4; parietal, P3/Pz/P4).

#### Basic memory effects

**Figure 2:**
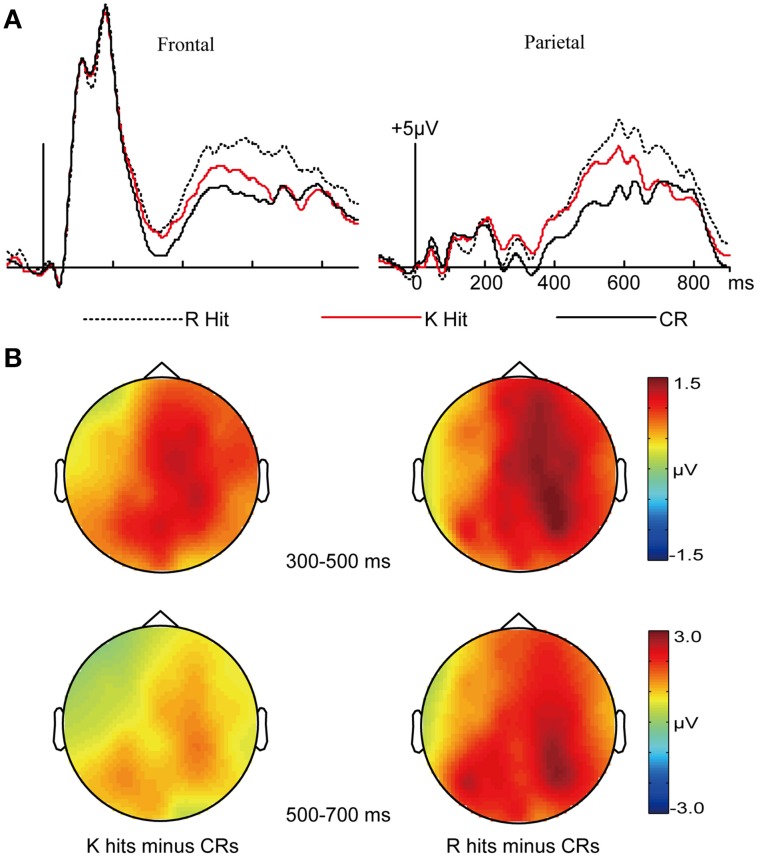
**ERPs related to recollection and familiarity**. ERPs related to recollection (*R* hits), familiarity (*K* hits), and correct rejections (CRs), collapsed across masked priming conditions. **(A)** Waveforms for each condition from frontal and parietal electrodes are shown. **(B)** Topographical plots depict ERP differences between *K* hits and CRs (left) and between *R* hits and CRs (right).

For the analysis of basic memory effects, *a priori* time windows of 300–500 and 500–700 ms were selected according to previous research (e.g., MacKenzie and Donaldson, 2007; Voss and Paller, 2007). These windows are fairly standard for the examination of FN400 and parietal old/new effects, respectively (Figure 2). The mean numbers of artifact-free epochs per condition were 120 for *R* hits, 110 for *K* hits, and 247 for CRs.

For the 300–500 ms interval, a 3 × 3 ANOVA was performed with factors response type (*R* hit/*K* hit/CR) and cluster (frontal/central/parietal). The main effect of response type was significant [*F*_(2, 30)_ = 9.44, *p* = 0.001, *MSE* = 2.065]. The interaction of response type and cluster was not significant [*F*_(4, 60)_ = 0.805, *p* = 0.456, *MSE* = 0.359]. Pairwise comparisons across the three conditions revealed significantly more positive amplitudes for *R* hits and *K* hits relative to CRs (*p* = 0.005 and 0.022, respectively), with no significant difference between *R* hits and *K* hits (*p* = 0.859).

For the 500–700 ms interval, a 3 × 3 ANOVA was performed with factors response type (*R* hit/*K* hit/CR) and cluster (frontal/central/parietal). The main effect of response type was significant [*F*_(2, 30)_ = 7.078, *p* = 0.007, *MSE* = 8.609]. The interaction of response type and cluster was not significant [*F*_(4, 60)_ = 2.367, *p* = 0.108, *MSE* = 0.507]. Pairwise comparisons across the three conditions revealed significantly more positive amplitudes for *R* hits relative to CRs (*p* = 0.021) and *K* hits (*p* = 0.042), with no significant difference between *K* hits and CRs (*p* = 0.3).

#### Masked conceptual priming effects

**Figure 3:**
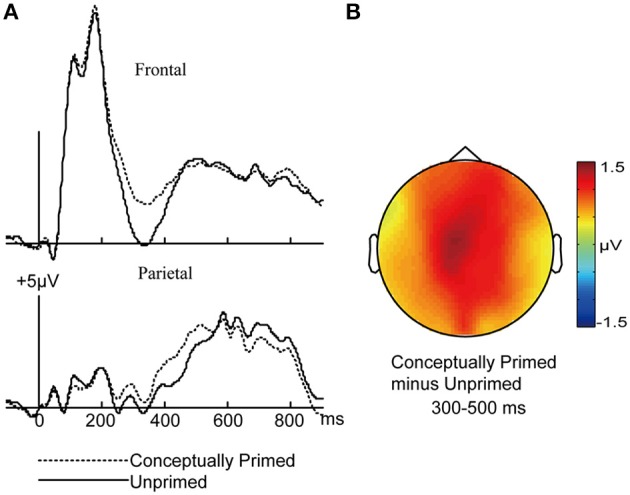
**ERP correlates of MCP and MUP test words**. ERPs to test words preceded by masked conceptually primes (MCP) and masked unrelated primes (MUP). **(A)** Waveforms from frontal and parietal electrodes are shown. **(B)** A topographical plot depicts ERP differences between MCP and MUP words.

First, the differences between MCP and MUP trials were investigated. The mean numbers of artifact-free epochs per condition were 282 for MCP trials and 285 for MUP trials. Amplitudes from 300 to 500 ms were more positive for MCP than for MUP trials (Figure 3), which is consistent with the typical latency of N400 (Kutas and Federmeier, 2011).

For the 300–500 ms interval, a 2 × 3 ANOVA was performed with factors masked conceptual priming (MCP/MUP) and cluster (frontal/central/parietal). The main effect of masked conceptual priming was significant [*F*_(1, 15)_ = 19.07, *p* = 0.001, *MSE* = 1.317]. The interaction of masked conceptual priming and cluster was not significant [*F*_(2, 30)_ = 0.99, *p* = 0.352, *MSE* = 0.181].

For the 500–700 ms interval, a similar 2 × 3 ANOVA was conducted. The main effect of masked conceptual priming was not significant [*F*_(1, 15)_ = 0.042, *p* = 0.84, *MSE* = 1.392]. The interaction of masked conceptual priming and cluster was not significant [*F*_(2, 30)_ = 0.055, *p* = 0.894, *MSE* = 0.14].

**Figure 4:**
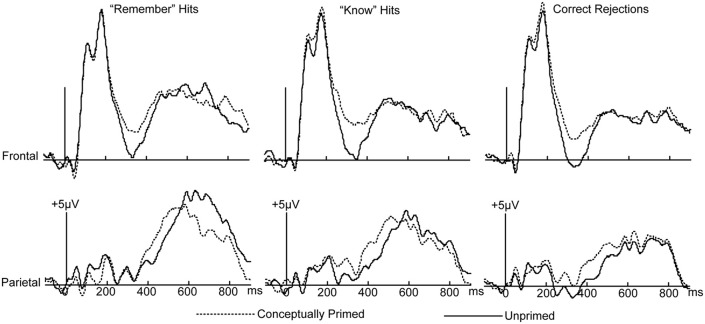
**Similarity of masked conceptual priming effects across three response types**. ERPs for *R* hits, *K* hits, and correct rejection trials as a function of MCP *vs.* MUP status. Waveforms from frontal and parietal electrodes are shown.

This priming effect was similar across the three response types (Figure 4). For each interval, a 2 × 3 × 3 ANOVA with factors masked conceptual priming (MCP/MUP), response type (*R* hit/*K* hit/CR), and cluster (frontal/central/parietal) was conducted. The mean numbers of artifact-free trials for *R* hits, *K* hits, and CRs were 62, 54, and 122, respectively, for MCP trials, and 58, 56, and 125, respectively, for MUP trials.

For the 300–500 ms interval, the main effects of masked conceptual priming and response type were significant [*F*_(1, 15)_ = 13.436, *p* = 0.002, *MSE* = 6.732; *F*_(2, 30)_ = 9.219, *p* = 0.001, *MSE* = 4.039, respectively], the main effect of cluster was not significant [*F*_(2, 30)_ = 0.286, *p* = 0.613, *MSE* = 83.48]. The interaction of masked conceptual priming and response type was not significant [*F*_(2, 30)_ = 0.701, *p* = 0.49, *MSE* = 1.743], the interaction of masked conceptual priming and cluster was not significant [*F*_(2, 30)_ = 0.951, *p* = 0.368, *MSE* = 0.738], the interaction of response type and cluster was not significant [*F*_(4, 60)_ = 0.87, *p* = 0.43, *MSE* = 0.712], and the three-way interaction was not significant [*F*_(4, 60)_ = 0.074, *p* = 0.953, *MSE* = 0.523].

For the 500–700 ms interval, the main effects of masked conceptual priming and cluster were not significant [*F*_(1, 15)_ = 0.254, *p* = 0.622, *MSE* = 5.405; *F*_(2, 30)_ = 0.402, *p* = 0.55, *MSE* = 73.817, respectively], but the main effect of response type was significant [*F*_(2, 30)_ = 7.061, *p* = 0.007, *MSE* = 17.012]. The interaction of masked conceptual priming and response type was not significant [*F*_(2, 30)_ = 2.062, *p* = 0.159, *MSE* = 4.757], the interaction of masked conceptual priming and cluster was not significant [*F*_(2, 30)_ = 0.094, *p* = 0.873, *MSE* = 0.764], the interaction of response type and cluster was not significant [*F*_(4, 60)_ = 2.477, *p* = 0.097, *MSE* = 1.024], and the three-way interaction was not significant [*F*_(4, 60)_ = 0.924, *p* = 0.421, *MSE* = 0.752].

**Figure 5:**
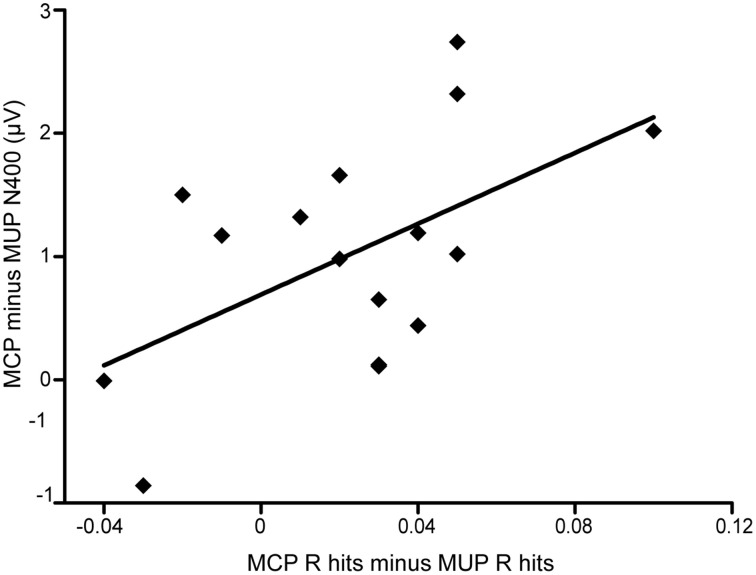
**Across-participant correlation between behavioral effect and ERPs difference**. Across-participant correlation between behavioral masked conceptual priming effect on *R* hits and difference in ERPs from 300 to 500 ms for MCP trials relative to MUP trials.

In complementary across-participant correlation analyses, we found that behavioral masked priming effects on *R* hits were related to N400 differences. This result provided tentative evidence for a connection between conceptual fluency–related ERPs and *R* responses. Many studies have used correlational analyses to examine associations between ERPs and behavior (e.g., Voss and Paller, 2006; Voss et al., 2010; Lucas et al., 2012) or among different ERP components (e.g., Meyer et al., 2007). Behavioral measures of masked priming effects were computed as: MCP *R*/*K* hits minus MUP *R*/*K* hits. ERP correlates of masked priming effects were calculated as the average amplitude differences of three electrode clusters between MCP and MUP trials from 300 to 500 ms. Across-participant correlations were thus computed between each behavioral measure of the masked priming effect separately for *R* hits and *K* hits, and ERP difference measures. For *R* hits, the behavioral measure of the masked priming effects was significantly correlated with the MCP /MUP amplitude difference (*n* = 16, *r* = 0.506, *p* = 0.046; Figure 5). For *K* hits, no significant correlation was found (*n* = 16, *r* = 0.307, *p* = 0.248).

## Discussion

The present study manipulated the conceptual fluency of recognition test cues and investigated the relationships between electrophysiological correlates of masked conceptual priming–induced fluency and recognition. Behavioral results indicated that the masked conceptual priming effect affected *R* hits, rather than *K* hits. A typical N400 effect was found when we compared the neural correlates of MCP and MUP items. In addition, across-participant correlation analyses revealed a significant relationship between the priming N400 effect and the priming effect on recognition memory for *R* hits, but not for *K* hits, suggesting that the N400 effects might be related to the effect of conceptual fluency on recollection. According to the fluency attribution account of recognition, these results suggest that recollection can be induced by conceptual fluency, which was derived from masked conceptual priming.

The results of our behavioral analysis replicated the key finding of Taylor and Henson (2012a) that masked conceptual priming could increase *R* hits. Importantly, participants in our experiment were exposed only to conceptual primes. In two previous studies (Taylor and Henson, 2012a; Taylor et al., 2013), the conceptual priming effect was observed only when conceptual and repetition primes occurred in the same experiment. Because they did not observe the conceptual priming effect in experiments that used only conceptual primes, Taylor and Henson (2012a) assumed that exposure to repetition primes might be a critical factor for conceptual priming effects on recollection. Taylor et al. (2013) proposed a “true” recollection account based on two critical findings: first, brain regions associated with genuine recollection were more active for conceptually primed trials than for unprimed trials; and second, the increase in *R* judgments following conceptual primes occurred only for studied items, but not for unstudied items.

We believe that our findings provide better support for the fluency-attribution account. First, in terms of the fluency-attribution framework, “old” responses are increased for more fluent (primed) items, irrespective of study status (e.g., Jacoby and Whitehouse, 1989; Rajaram, 1993). In other words, fluency manipulation tends to increase both hits and FAs. In the present study, masked conceptual priming increased not only *R* hits, but also *K* FAs. Second, the observed association between conceptual priming and N400-like ERP potentials also provides better support for the fluency-attribution account than for the “true” recollection account. These findings suggest that the mechanism behind these effects was processing fluency.

Intriguingly, we found conceptual priming effects on recollection that Taylor and Henson (2012a) did not find when only conceptual prime blocks were used, although we used a similar paradigm. We contend that memory performance might play a key role in this difference. Compared with their findings, our sample showed higher overall accuracy, but a lower recollection ratio, which might lead to distinct fluency effects on recollection. Another possible reason for the discrepancy in results is our use of different stimuli (Chinese words *vs*. English words in Taylor and Henson, 2012a). Further studies should be conducted to investigate this phenomenon in greater depth.

Most studies of the relationship between conceptual fluency and recognition memory have provided support for the influence of conceptual fluency on familiarity (Rajaram and Geraci, 2000; Wolk et al., 2004). In addition, a nearly ubiquitous complication of the use of the R/K paradigm to distinguish recollection from familiarity is that it can be very difficult to tell whether *R* and *K* responses are used merely to separate strong memories from weak memories, or whether participants actually map them onto experiences of recollection and familiarity *per se*. *R* responses may have been confounded to some extent by familiarity in the present study. These concerns could be alleviated by the following two points. First, when providing instructions, the experimenter emphasized clearly that participants could not equate *R*/*Familiar* responses with high/low confidence ratings. To ensure that they understood these instructions correctly, participants were asked to report the reasons for *R* and *K* responses in the practice blocks. Some studies have suggested that such instructions help to prevent participants from confusing R/K instructions with confidence instructions (Yonelinas, 2001; Rotello et al., 2005; Yonelinas and Parks, 2007). Second, in the analysis of basic memory effects, ERP differences between *K* hits and CRs emerged in the 300–500 ms interval, and those between *R* hits and CRs emerged in both intervals. These results suggest that the timing of neural activity associated with *R* and *K* responses differs. Therefore, *R* responses obtained in the present study should reflect recollection.

When we collapsed ERPs across response type and old/new status to examine masked conceptual priming effects, a typical N400 effect was obtained. Amplitudes from 300 to 500 ms were more positive for MCP than for MUP trials. This effect was found in most previous studies investigating the effect of masked repetition priming on recognition (Woollams et al., 2008; Lucas et al., 2012). More broadly, studies investigating masked repetition-priming effects also identified a similar N400 effect (Deacon et al., 2000; Holcomb and Grainger, 2006). However, whether masked conceptual (semantic) priming elicits such an N400 effect remains under debate. For instance, some studies (Kiefer, 2002; Kiefer and Brendel, 2006) obtained the N400 effect when investigating masked semantic (conceptual) priming. In contrast, Brown and Hagoort (1993) observed a significant N400 effect under the unmasked, but not the masked, presentation condition. The results of the present study support the idea that masked conceptual priming can induce N400 effects. Moreover, these N400 semantic priming effects were dependent on the activation of primes, although they were subliminal.

This priming effect was similar across the three response types (*R* hit/*K* hit/CR). In addition, across-participant correlation analyses found that participants who showed larger N400 differences between MCP and MUP trials also showed greater increases in *R* hits for MCP compared with MUP trials. These results suggest that the N400 effect was associated with the contribution of conceptual fluency to recollection recognition. However, some scholars have suggested that familiarity and recollection are correlated with the FN400 old/new effect and LPC, respectively (e.g., Rugg and Curran, 2007), whereas other researchers have argued that FN400 potentials are associated with conceptual priming (e.g., Paller et al., 2007). To reconcile this controversy, one can resort to the relationship between recognition memory and fluency (Lucas et al., 2012; Lucas and Paller, 2013). For example, FN400 potentials may reflect a conceptual fluency–related precursor to familiarity (Lucas et al., 2012). Further investigations should be done to examine the relationship between conceptual fluency and recognition memory.

Most previous studies have suggested that conceptual fluency can give rise to familiarity. For example, a series of studies by Wang and colleagues (Wang et al., 2010, 2014; Wang and Yonelinas, 2012a,b) showed that the conceptual fluency induced by conceptual priming can result in the feeling of familiarity. Moreover, some studies have suggested that familiarity judgments can also stem from the perceptual fluency induced by perceptual priming (e.g., Rajaram, 1993; Lucas and Paller, 2013). However, the results of the present study suggest that conceptual fluency is related to recollection. These findings indicate that the contribution of fluency to recognition is complex and not necessarily characterized by one-to-one correlation between the types of fluency and the types of recognition process. Thus, additional studies are needed to identify factors that determine how fluency translates into recognition under various testing conditions.

Many years ago, Mayes et al. (1997) proposed that experiences of recollection could result partially from the attribution of fluency to prior experience. They posited that one could readily confuse an imagined with a remembered item-context association. However, a variety of factors can affect the extent to which a given amount of fluency is attributed to and experienced as recollection, which requires further study. We cannot rule out the possibility that conceptual priming effects may be “artifacts” of the binary (either/or) nature of the R/K procedure. In other words, one might not expect to find such an effect if one used the parallel R/K ratings procedure (Higham and Vokey, 2004; Kurilla and Westerman, 2008; Brown and Bodner, 2011), added a third option (e.g., guesses, Tunney and Fernie, 2007), allowed indication of different types of recollection (e.g., internal *vs*. external source), or minimized recollection using the modified R/K procedure (e.g., Montaldi et al., 2006). Taken together, these concerns require new, robust evidence provided by behavioral and neural data.

### Conflict of interest statement

The authors declare that the research was conducted in the absence of any commercial or financial relationships that could be construed as a potential conflict of interest.
