# Supplementary material for: Conceptual fluency increases recollection: behavioral and electrophysiological evidence
Source: Front Hum Neurosci. 2015 Jun 30;9:377. doi: 10.3389/fnhum.2015.00377 (PMC4485059; doi:10.3389/fnhum.2015.00377)
Supplement: Supplementary file 1 [file DataSheet1.DOCX]

蝶泳 蛙泳 灯具 台灯 海豚 鲸鱼 书橱 书架

贺词 贺电 老鹰 雄鹰 囚车 押解 鹦鹉 八哥

生辰 生日 羚羊 斑马 赏脸 赏光 逞能 逞强

端午 粽子 道士 道教 干妈 干娘 保镖 护卫

米酒 白酒 钓鱼 钓竿 露营 帐篷 钱包 钱币

伴唱 伴奏 画板 画布 花苞 花瓣 草书 行书

偷窃 偷盗 补品 补药 房檐 屋檐 分针 秒针

帮会 帮派 晚宴 餐具 尸骸 尸骨 跳蚤 虱子

考场 考卷 马桶 厕所 秤杆 秤钩 棒球 网球

苦工 苦力 行刺 刺客 囚犯 囚徒 炒面 炒米

药瓶 药片 欠债 负债 彩霞 彩云 电扇 风扇

菠萝 芒果 牵挂 挂念 共享 分享 伤兵 伤员

后代 后人 日历 台历 戒指 项链 鬼怪 幽灵

水星 土星 尸身 尸首 骑士 盾牌 孤单 孤独

绑架 绑票 袈裟 僧人 湖水 湖泊 早餐 早点

猪蹄 猪头 搜寻 搜索 抚慰 安慰 宝藏 宝物

隐士 隐居 头疼 头痛 药铺 药材 冬至 夏至

豪放 豪爽 庭院 院子 镜片 墨镜 进贡 贡品

猩猩 猴子 耳环 耳垂 怯懦 胆怯 拱桥 桥梁

乌龟 蜗牛 牙膏 牙刷 冬瓜 南瓜 城堡 堡垒

侄子 侄女 薪金 薪水 主角 主演 惦记 惦念

枫树 枫叶 营地 军营 陆路 水路 水饺 饺子

头衔 称号 前院 后院 谎话 谎言 旗袍 裙子

喜酒 喜事 盆景 盆栽 邮票 信封 毛衣 毛线

圆桌 方桌 除夕 春节 憧憬 向往 金币 银币

禽兽 畜生 字典 词典 飞船 太空 伴侣 配偶

浴室 洗澡 皱眉 气愤 家产 家当 处方 药方

孔雀 凤凰 甜瓜 苦瓜 假期 假日 玉器 玉石

讲台 教室 随从 跟班 兵书 兵法 衡山 嵩山

郊外 郊区 明星 名人 伤疤 伤痕 城楼 城门

汉堡 西餐 拍照 照相 海鸥 海燕 滑冰 滑雪

军服 军装 树梢 树枝 竹竿 竹叶 魔术 魔法

雇员 雇主 眼眶 眼角 焦虑 忧虑 飓风 台风

津贴 补贴 暑假 暑期 梳子 梳头 群岛 岛屿

堤坝 水坝 阅兵 检阅 晚霞 朝霞 橘子 香蕉

鞭打 抽打 前门 后门 麻雀 燕子 量筒 烧杯

口吃 结巴 胶带 胶布 沼泽 泥沼 诗集 诗篇

毛笔 书法 病房 病床 要饭 讨饭 战俘 战犯

额头 皱纹 疲乏 疲倦 退伍 复员 水晶 宝石

幸运 走运 习俗 风俗 书信 信件 暖气 供暖

栅栏 篱笆 香油 芝麻 除号 乘号 员工 老板

棉衣 棉被 妓女 妓院 国君 皇上 桃树 桃花

兔子 兔毛 荷花 莲花 杂技 杂耍 店铺 店员

汉语 汉字 章节 篇章 精华 精髓 媒人 媒婆

砖头 石板 婚礼 新娘 针尖 针头 猜想 猜测

糖果 软糖 武术 刀枪 样式 款式 工匠 瓦工

句子 短语 火炮 大炮 瘦弱 瘦小 报馆 报社

榆树 杨树 饮料 果汁 苍蝇 蚊子 绿地 绿化

声调 音调 浇灌 浇水 头骨 颅骨 悲伤 难过

书房 书桌 使者 使臣 玩具 皮球 船长 水手

火山 岩浆 南亚 东亚 链球 标枪 地狱 魔鬼

公告 布告 歌剧 话剧 绿豆 红豆 生病 患病

象棋 棋子 勤奋 勤勉 北极 南极 英语 德语

虐待 侮辱 烦恼 苦恼 元帅 将领 手绢 手帕

咽喉 喉咙 刀子 匕首 证物 证词 乘客 客船

医师 护士 礼物 礼品 木工 木匠 演讲 讲座

海港 港湾 镇静 平静 佃户 佃农 手枪 子弹

枝叶 枝干 史书 史籍 创建 创立 获胜 取胜

讲课 授课 犯人 罪犯 铅球 铁饼 日出 黎明

歌词 歌谱 旅馆 旅店 打扮 化妆 胭脂 脂粉

英镑 法郎 嘲笑 嘲讽 巡逻 巡警 野兽 野猪

降水 降雨 垃圾 废品 当兵 服役 灾区 灾民

山谷 峡谷 推迟 延迟 男孩 女孩 鼓掌 掌声

手表 怀表 讽刺 讥讽 稻田 稻子 骗子 骗人

老虎 狮子 谦虚 谦逊 参军 入伍 皮鞋 凉鞋

卡车 货车 白糖 红糖 白菜 萝卜 花朵 鲜花

毒品 白粉 大米 米饭 赞美 赞赏 丝绸 绸缎

步兵 骑兵 处死 处决 钢琴 吉他 饭店 饭馆

清晨 早上 蜡烛 烛光 坏人 坏蛋 官府 衙门

牧场 牧草 模仿 模拟 味精 料酒 沙发 家具

监狱 监牢 面孔 面容 助手 助理 饥饿 饥荒

废料 废物 草地 草坪 排练 彩排 衣裳 外套

口袋 麻袋 雕塑 雕刻 镜头 相机 馒头 面食

炸药 火药 刊物 月刊 潮流 时尚 胡说 胡扯

面包 糕点 荣誉 荣耀 种地 种田 食指 拇指

烧鸡 烤鸭 槐花 槐树 古玩 古董 浴场 浴池

住持 方丈 烤肉 烤炉 月饼 中秋 海啸 海浪

肉铺 猪肉 长笛 横笛 彩绸 彩带 瞳孔 眼球

金条 金子 菜谱 菜单 土炕 炕头 名望 声望

茶花 茶树 古琴 古筝 解聘 解雇 电脑 主机

猎犬 猎狗 生肖 属相 桥洞 桥身 虾仁 虾皮

瓦片 瓦砾 妒忌 嫉妒 车胎 轮胎 汤勺 汤匙

丝袜 袜子 彗星 流星 邮包 邮件 坚果 核桃

算卦 算命 计谋 计策 拐杖 拐棍 赃款 赃物

谷仓 粮仓 卖唱 卖艺 竹篓 竹筐 小费 侍者

战机 战舰 果仁 果肉 矿长 矿工 犀牛 河马

球鞋 胶鞋 传言 传闻 邻里 街坊 蚂蚱 蟋蟀

棺木 棺材 大雁 鸿雁 船舷 船舱 车灯 车门

冰棒 冰糕 奏章 奏折 舞女 舞厅 合影 照片

染缸 染坊 煤球 煤炉 野兔 家兔 海龟 海豹

钟楼 鼓楼 账目 账簿 猎人 猎手 白薯 红薯

台词 对白 密码 口令 烟花 礼花 施舍 救济

笔迹 字迹 摔跤 柔道 天台 楼顶 富翁 富豪

汤圆 元宵 印章 图章 冲撞 撞击 泥沙 泥土

勾引 勾搭 柳枝 柳树 雪茄 香烟 故土 故里

秋千 吊环 镜框 眼镜 空降 空投 后母 继母

墙头 墙角 横幅 条幅 害羞 腼腆 名册 名单

小溪 溪流 酒席 酒菜 证券 股票 晨报 晚报

电梯 楼梯 床单 被褥 插头 插座 门框 门槛

管家 仆人 春光 春色 故人 故交 人参 燕窝

砒霜 毒药 裤脚 裤腿 容貌 相貌 扶手 把手

试卷 试题 恭喜 恭贺 丢脸 丢人 麦粒 麦穗

污浊 污水 雨林 丛林 海棠 杜鹃 说谎 撒谎

日食 月食 锅底 锅盖 触角 触须 战地 战车

迁徙 候鸟 轿子 轿夫 冰箱 冷藏 钻石 珍珠

冰糖 奶糖 灯笼 灯谜 判官 阎王 词汇 单词

叛国 叛变 仇人 仇敌 浴缸 澡盆 电焊 焊工

番茄 葡萄 大蒜 洋葱 特工 间谍 感冒 咳嗽

讲师 教员 腰带 腰部 香皂 洗手 启程 上路

绰号 外号 邮局 邮递 喜鹊 乌鸦 枪支 步枪

绿洲 荒漠 洞穴 洞口 拘留 拘役 背心 短裤

表白 告白 海滩 海岸 羊肉 牛肉 爬山 登山

祈祷 祷告 茶壶 茶具 英尺 英寸 话筒 听筒

裁缝 裁剪 寺庙 僧侣 捐献 捐赠 微风 狂风

开心 快活 声母 韵母 学识 学问 蛋黄 蛋清

凳子 板凳 衬衣 衬衫 矿藏 矿产 炉灶 炉火

奖章 奖杯 茄子 黄瓜 学士 硕士 饼干 零食

侨胞 侨民 胜负 成败 手心 手背 伐木 砍伐

封面 封皮 露水 露珠 蛤蟆 蟾蜍 圈套 陷阱

海边 海滨 喜剧 悲剧 古籍 古书 枕头 枕巾

打猎 狩猎 尘埃 尘土 坟墓 墓碑 过错 过失

剧院 剧场 拜访 拜见 更改 更正 呼喊 呼叫

枯萎 凋谢 烧饼 馅饼 太监 宦官 跳舞 舞会

背包 书包 大雪 小雪 青蛙 水塘 江湖 武林

卧室 床铺 手艺 技能 乞丐 乞讨 水灾 水患

旱灾 旱情 中药 草药 算盘 珠算 功劳 功臣

铁轨 钢轨 散文 诗歌 斥责 训斥 牡丹 茉莉

喉头 喉结 山峰 山顶 油污 油渍 跳水 跳台

脚掌 脚趾 光棍 寡妇 稻谷 稻米 奴才 奴仆

墨水 墨迹 骆驼 牦牛 理发 剃头 躯干 躯体

樱桃 草莓 罕见 稀有 黑板 粉笔 山羊 绵羊

皇室 皇族 目录 清单 耕牛 耕田 报表 表格

袖子 袖口 桑树 桑叶 穷苦 穷困 楼房 平房

蝴蝶 蜜蜂 回民 回族 静脉 动脉 竹子 竹笋

老鼠 耗子 声势 气势 学徒 师父 积聚 积蓄

责备 责怪 惩办 惩处 鲫鱼 鲤鱼 短跑 长跑

包袱 包裹 大雨 小雨 海峡 海湾 耽误 耽搁

键盘 屏幕 牧民 牧区 地板 地毯 胖子 肥胖

河床 河道 景色 景观 炮兵 炮手 真相 实情

大红 粉红 噪声 噪音 马车 车夫 红茶 绿茶

同伴 同伙 大腿 膝盖 丞相 宰相 林场 林地

方形 菱形 黄昏 日落 军火 坦克 图画 素描

测试 测验 晚饭 午饭 炸弹 弹药 奇数 偶数

推测 揣测 行李 皮箱 葬礼 花圈 渔船 渔夫

信念 信仰 家族 族长 味道 香甜 油画 版画

氮肥 钾肥 亲戚 亲属 标点 句号 户口 户籍

帮忙 帮手 喇叭 汽笛 直尺 文具 影片 影院

盗窃 盗贼 藏族 藏民 窗户 窗帘 玫瑰 月季

家人 姐妹 师生 师徒 风景 风光 初中 高中

跳高 跳远 造反 反叛 乘法 除法 火箭 导弹
